# Modelling the relative abundance of the primary African vectors of malaria before and after the implementation of indoor, insecticide-based vector control

**DOI:** 10.1186/s12936-016-1187-8

**Published:** 2016-03-05

**Authors:** Marianne E. Sinka, Nick Golding, N. Claire Massey, Antoinette Wiebe, Zhi Huang, Simon I. Hay, Catherine L. Moyes

**Affiliations:** Spatial Ecology and Epidemiology Group, Department of Zoology, University of Oxford, Oxford, OX1 3PS UK; Wellcome Trust Centre for Human Genetics, University of Oxford, Oxford, OX3 7BN UK; Institute for Health Metrics and Evaluation, University of Washington, Seattle, WA 98121 USA; Fogarty International Center, National Institutes of Health, Bethesda, MD 20892-2220 USA

**Keywords:** Relative abundance, Vector, Mosquito, *Anopheles*, Africa, Insecticide control, Malaria

## Abstract

**Background:**

Malaria remains a heavy burden across sub-Saharan Africa where transmission is maintained by some of the world’s most efficient vectors. Indoor insecticide-based control measures have significantly reduced transmission, yet elimination remains a distant target. Knowing the relative abundance of the primary vector species can provide transmission models with much needed information to guide targeted control measures. Moreover, understanding how existing interventions are impacting on these relative abundances highlights where alternative control (e.g., larval source management) is needed.

**Methods:**

Using the habitat suitability probabilities generated by predictive species distribution models combined with data collated from the literature, a multinomial generalized additive model was applied to produce relative abundance estimates for *Anopheles arabiensis, Anopheles funestus* and *Anopheles gambiae/Anopheles coluzzii*. Using pre- and post-intervention abundance data, estimates of the effect of indoor insecticide-based interventions on these relative abundances were made and are illustrated in post-intervention maps.

**Results:**

Conditional effect plots and relative abundance maps illustrate the individual species’ predicted habitat suitability and how they interact when in sympatry. *Anopheles arabiensis* and *An. funestus* show an affinity in habitat preference at the expense of *An. gambiae*/*An. coluzzii,* whereas increasing habitat suitability for *An. gambiae*/*An. coluzzii* is conversely less suitable for *An. arabiensis* but has little effect on *An. funestus.* Indoor insecticide-based interventions had a negative impact on the relative abundance of *An. funestus,* and a lesser effect on *An. arabiensis*. Indoor residual spraying had the greatest impact on the relative abundance of *An. funestus,* and a lesser effect on *An. gambiae*/*An. coluzzii*. Insecticide-treated bed nets reduced the relative abundance of both species equally. These results do not indicate changes in the absolute abundance of these species, which may be reduced for all species overall.

**Conclusions:**

The maps presented here highlight the interactions between the primary vector species in sub-Saharan Africa and demonstrate that *An. funestus* is more susceptible to certain indoor-based insecticide interventions than *An. gambiae*/*An. coluzzii*, which in turn, is more susceptible than *An. arabiensis*. This may provide *An. arabiensis* with a competitive advantage where it is found in sympatry with other more endophilic vectors, and potentially increase the need for outdoor-based vector interventions to deal with any residual transmission barring the way to malaria elimination.

**Electronic supplementary material:**

The online version of this article (doi:10.1186/s12936-016-1187-8) contains supplementary material, which is available to authorized users.

## Background

The primary malaria vectors in Africa are *Anopheles gambiae* sensu stricto (now *Anopheles gambiae* and *Anopheles coluzzii*) [1], *Anopheles funestus* and *Anopheles arabiensis*. *Anopheles gambiae/An. coluzzii* are members of the *An. gambiae* complex and are considered to be the most efficient malaria vectors in existence [2]. *Anopheles funestus* is thought to have been the first African species to exploit humans as a food source [3] and is regarded as a more efficient vector than *An. gambiae*/*An. coluzzii* in some parts of its range [4]. *Anopheles arabiensis* is another member of the *An. gambiae* complex and is considered less anthropophilic than either *An. gambiae, An. coluzzii* or *An. funestus* [5]. It tends to maintain a life cycle outdoors and is thus more likely to avoid the two primary methods of vector control used across Africa (long-lasting insecticidal nets (LLINs) and indoor residual spraying (IRS)) than the other species [6]. This allows malaria transmission to continue even where the abundance of the other two species has been significantly lowered [7]. A measure of relative abundance is a simple and clear indication that further control may be required. A sudden shift in the relative abundance within a targeted group of species after the implementation of a control programme illustrates a hierarchy of effectiveness of the control. It can also indicate emerging behavioural or physiological insecticide resistance. By estimating the relative abundance of multiple species in a location, predictions can be made about the continuing impact of specific control measures where the species’ biology or behaviour are known [7].

Clearly a measure of ‘true’ or ‘absolute’ abundance confers valuable information but abundance data collated across time and space are notoriously fraught with bias; mosquito species densities are highly heterogeneous, showing year–year or seasonal variability (e.g., before or after rains) and within small scale space (e.g., near or far from productive larval sites) [8]. Trying to combine abundance data from more than one source to create a spatially diverse dataset is essentially impossible due to variability in the methods of capture and sampling effort. Relative abundances, the proportion of the mosquito population belonging to each species, however, are more easily comparable and are less likely to be affected by these sources of bias, keeping in mind the caveat that certain sampling methods may favour some species over others. Relatively robust predictions of relative abundance can be made from diverse data sources that cannot currently be achieved for ‘true’ abundance.

Relative abundance is specifically useful for malaria transmission models that incorporate species-specific vector parameters (such as larval site characteristics, biting habits and resting behaviour) [9]. Such models often include the entomological inoculation rate (EIR), which can vary depending on the species composition in a given area. The EIR of a species is dependent on human biting rate (HBR: the number of bites per human per unit time), which is indicative of abundance/density, and the sporozoite rate (the number of sporozoite-positive specimens over the total collected). The EIR can also be extrapolated back from estimates of parasite rate [10, 11] but this does not provide detail on the vector species responsible for transmission. With HBR being highly species-specific and strongly influenced by density, relative abundance estimates help bridge the gap left by sparse EIR data allowing existing transmission models to be applied over space and time.

Whilst presence-only species distribution models (SDMs) are used to predict the relative probability of presence of a species, rather than abundance, both metrics are likely to be positively related to habitat suitability. Such a monotonically increasing relationship between abundance and suitability has been identified in a range of taxa [12, 13] and has been used to relate disease prevalence to habitat suitability [14].

This relationship is exploited here by using maps of habitat suitability for anopheline species [15] and field data on abundance prior to the introduction of interventions to generate spatial predictions of the relative abundance of the primary African malaria vectors. Estimates of the effect of the main insecticide-based control interventions: LLINs and IRS on the relative abundances of these species are also given.

## Methods

### Habitat suitability maps

A previous study mapping the global distribution of the dominant vector species (DVS) of malaria, including seven species across Africa [15], involved a comprehensive literature search to identify and collate occurrence data for each species. This resulted in the creation of a unique and comprehensive database of global, contemporary (post 1985–2010) vector occurrence (data are available via [16]). These data underwent an in-depth checking procedure, which included being assessed by a technical advisory group of vector experts who also helped to create expert opinion range limit maps for each species. Using the boosted regression tree (BRT) niche modelling methodology [17] combined with remotely sensed climatic and environmental covariate data, habitat suitability maps (represented as the relative probability of presence at every 5 × 5 km pixel) were created for each species (more detail on the climatic parameters behind the habitat probabilities are given in Additional file 1: Table S1, and a full methodology is given in [15]).

The relative probabilities of presence estimated by these models are not directly comparable across different species. For example, a low probability for one species in a pixel and a high probability of a second species in the same pixel will not automatically indicate that the latter will be found in higher densities, as there are many other biological factors that come into play. The natural carrying capacity of a highly suitable location could be much lower for one species compared to another due to ecological factors such as predation, competition or the density of accessible hosts or larval sites. Thus, additional analysis is required to understand the relationship between these habitat suitability measures and relative abundances found in the field.

### Relative abundance data

During the process of collating data for the occurrence mapping study described above, additional details were recorded. For example, the methods by which the mosquitoes had been sampled was recorded, and, where given, the numbers of individual mosquitoes from each species captured by each reported sampling method, giving comparable species-specific abundance data at a given time and location. The sampling methods included indoor (e.g., indoor resting, indoor biting, indoor light traps) as well as outdoor (e.g., outdoor resting in shelters or vegetation, outdoor biting) methods using both human (e.g., human-baited nets) and animal (e.g., animal-baited nets) attractants. Certain behavioural characteristics of the mosquito species will cause a bias in numbers collected by different sampling methods. *Anopheles arabiensis* is considered to be considerably less endophagic (indoor biting) then either *An. funestus* or *An. gambiae*/*An. coluzzii*. Therefore any study using indoor human landing catches may under-represent the presence of *An. arabiensis* in the study area. However, the data used here included those from a combination of different studies and a variety of sampling methods. As such, some of the inherent bias will have been mitigated in the relative abundance estimates. Data were pulled from the database that gave sample numbers for two or more of the seven DVS in Africa using the same sampling method during the same sampling period at the same study site. Latterly, due to limited data for some species, the analysis was refined to include only *An. arabiensis*, *An. funestus* and *An. gambiae*/*An. coluzzii.* Data were collated from the more recent literature, and these were added to the DVS database.

### Intervention data

The DVS database also records the presence of chemical vector control, such as LLINs or IRS. Therefore, searches for studies that specifically detailed the effects of such vector control on the densities of African DVS were made. To ensure the dataset was as inclusive and up to date as possible, additional searches were conducted within the more recent literature (PubMed and Web of Science) for data published from 2010 onwards. Each published source was examined and data abstracted for comparable measures of intervention and non-intervention population samples for the primary African DVS. Although the data included longitudinal studies (where the confounding factor may be variability in season/weather conditions) and spatial data (where confounding factors include variability in spatial characteristics such as proximity to larval sites that could impact on species abundance), only the spatial studies were used in the final analysis as a greater number of these were found in the literature. Moreover, the longitudinal data were compromised by the variability in the length of time between sampling events across studies; should the model examine the initial impact of the interventions or look at more long term effects? Of the spatial studies, only those where effort had been made to limit the confounding factors were included. Additionally, only those studies that used the same sampling methods and effort for the intervention and non-intervention measures were included.

### Modelling relative abundance

Using only those pixels where a probability of presence was estimated to be greater than 0.5 for all species, the predicted habitat suitability for each species at the geo-referenced location of each record was extracted from the habitat suitability data layers. These data were then used to fit a multinomial generalized additive model (GAM) using the VGAM R package [18], using the number of individuals of each species as the response and the predicted habitat suitability indices for each species in the sub-group as predictors.

The flexibility of the response curves fitted by the GAM is controlled by a positive degrees of freedom parameter k, with k = 1 corresponding to linear terms and higher values of k allowing more complex non-linear responses. Whilst increasing the value of k increases the fit of the model to the training data, using too high a k causes the model to overfit to the training data and predict new data poorly. In order to identify an optimal value of k for the spatial modelling task, a spatially stratified, cross-validation procedure was carried out. First, the 141 relative abundance data occurrences were partitioned into 20 clusters of spatially adjacent points by k-means clustering on the coordinates of the study site. In order to find clusters with short distances between component datapoints, but with comparable numbers of datapoints in each group, the clustering algorithm was run 1000 times and selected the clustering with the smallest mean absolute pair-wise difference in cluster sizes. The spatially stratified, out-of-sample predictive power of the GAM models were evaluated with different values of k by fitting each candidate model 20 times, each time withholding one of the 20 clusters. The total negative likelihood of the withheld data, given the predicted abundance ratios for each cluster, were summed to calculate the overall validation metric. This test was run for 30 different values of k, evenly spaced between 1 and 3. This resulted in a clear concave curve, with a minimum (the value of k giving the most accurate prediction) at 2.24, which was used to fit the final model.

The fitted GAM was then used to predict the relative abundance of each species (as a proportion of the total abundance of the species combined) at each pixel. In order to map uncertainty in these estimates, the dataset was bootstrap re-sampled 100 times and a multinomial GAM fitted to each bootstrap using the fixed value of k and the 95 % confidence intervals for the predicted relative abundance calculated.

### Modelling intervention effects

Estimates of the effect of indoor-based insecticide control measures on the abundance of the three vector species were made by fitting a generalized linear model with Poisson likelihood and logarithmic link via maximum likelihood inference. Observed abundance in the intervention sample was considered as the dependant variable and the observed log-abundance in the control sample used as an offset in the linear component. Consequently, the remainder of the linear component modelled the expected log-ratio between intervention and control populations, providing an estimate of the impact of the intervention on abundance. Regression terms for each of the species and intervention types were included as well as interactions between them, enabling the model to pool information across all available data whilst allowing for species- and intervention-level differences in efficacy. A regression term for whether sampling was carried out by collecting mosquitoes resting indoors was also included.

Models were fitted with each of these regression terms (species, intervention, species-intervention interaction, and household sampling) in turn and the models were compared by Akaike information criterion value (AIC) and likelihood ratio tests to determine the importance of each predictor. Using the initial model, the expected change in relative abundance for each species-intervention combination was predicted. In order to quantify uncertainty in these predictions, 10,000 realizations of the expected intervention/control ratio estimates for each species-intervention combination from the joint profile likelihood of the model were sampled.

The uncertainty in the prediction from this model was propagated into uncertainty in the predicted post-intervention relative abundance maps by selecting 100 of these reduction rate estimates and pairing each with one of the bootstrapped pre-intervention relative abundance maps to generate realisations of post-intervention relative abundances for the three species. All models were fitted using R version 3.2.1 [19].

## Results

The final analysis for relative abundance used only those data where adult abundance of all three species were reported (including ‘0’ abundances) and where members of the *An. gambiae* complex were identified to species. Studies where pre-existing control measures were in place were removed so the data represented relative abundance before the influence of chemical interventions. Once these exclusions were implemented, the remaining data consisted of 38,067 individual mosquitoes (12,646 *An. arabiensis*, 12,103 *An. funestus* and 13,318 *An. gambiae*/*An. coluzzii*) from 141 collections at 51 sites from 31 published sources (see Additional file 1: Figure S1 illustrates the spread of data across the continent). A total of 51 sites across Africa is a small dataset spread over a large area, a factor that needs to be considered when interpreting the modelled results.

Sources reporting ‘0’ densities post-control were removed from the ‘intervention’ data as these tended to represent studies reporting initially low vector densities prior to the application of the intervention. The resulting, final ‘post-intervention’ dataset was also small, with 21 sources providing 575 measurements of spatially comparable pre and post-intervention species densities. These data spanned both wet and dry seasons with eight covering both, 265 in the dry season and 294 in the rainy season. Data covered west (Benin, Burkina Faso, The Gambia, and Nigeria), east (Kenya, Ethiopia, Sudan, and Tanzania) and south (Madagascar, Mozambique and South Africa) Africa.

### Pre-intervention relative abundance

**Fig. 1:**
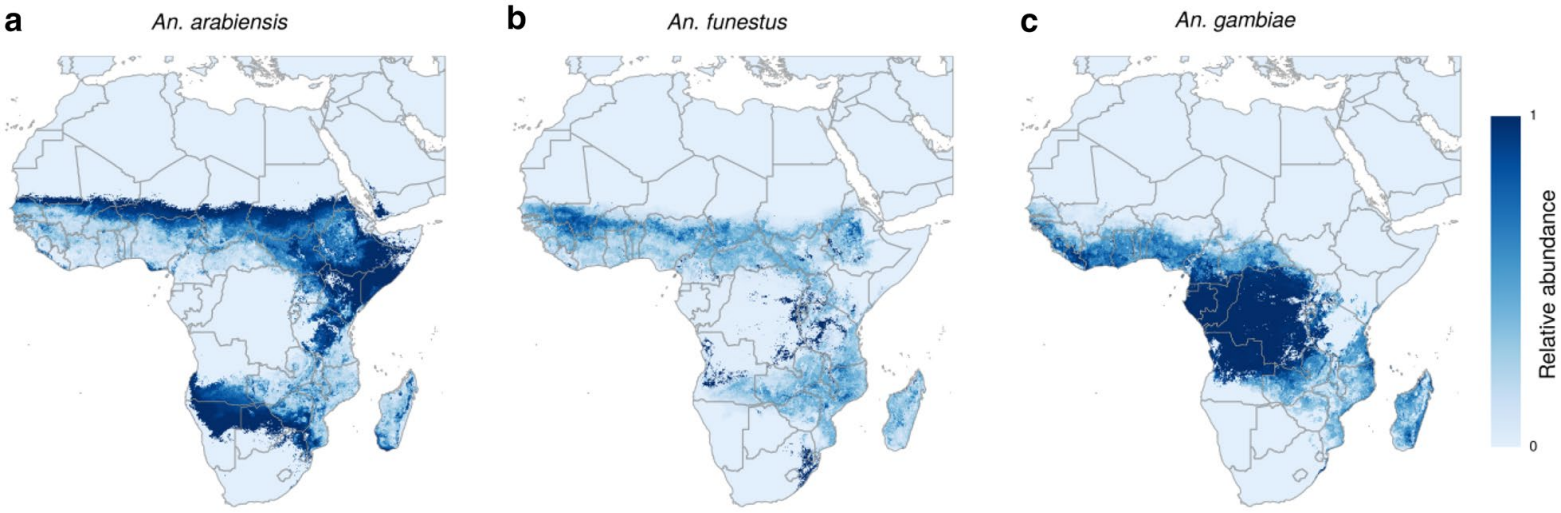
Relative abundance of each vector species predicted by the multinomial generalized additive model (no control). Plot **a** shows *An. arabiensis*, plot **b** shows *An. funestus* and plot **c** shows *An. gambiae*/*An. coluzzii*. The relative abundances for each cell across all three maps sums to one where any vector species is present, or zero where all species are absent. The extent of the predictions are restrained to the probability of presence extents as predicted by the BRT outputs [15], where probability of presence ≥0.5. These, in turn were restricted in the original maps by masking the predictions at a buffer of 1500 km. When interpreting these maps, note they do not show absolute abundance. For example, despite a clear presence of *An. funestus* on the northern fringes of the Sahel, the maps are showing it is more abundant than both *An. arabiensis* and *An. gambiae* but not that it is found in abundance. Conversely, the *An. funestus* map shows that it is *less abundant* than *An. gambiae* in central Africa, not that it does not exist here (*An. gambiae* and *An. funestus* are both highly efficient and dangerous vectors in central Africa)

The multinomial GAM of relative abundance explained 49.6 % of deviance in a multinomial intercept-only null model. Figure 1 maps the *relative* abundances of each species as predicted by this model. There appears to be a fairly clear ‘preferred’ environment for each species even where they are sympatric. *Anopheles arabiensis* (Fig. 1a) dominates in the drier northern and southern reaches of sub-Saharan Africa whereas travelling inwards towards central Africa, *An. funestus* (Fig. 1b) bridges the gap (but remains less comparably abundant then either of the other two species) until the dominance of *An. gambiae*/*An. coluzzii* emerges (Fig. 1c), centred in the more humid forested areas of central and western Africa. Again, this is not showing an abundance of *An. gambiae*/*An. coluzzii* but more likely a reduced relative abundance of *An. arabiensis* (not considered to be present in these areas) and *An. funestus*.

**Fig. 2:**
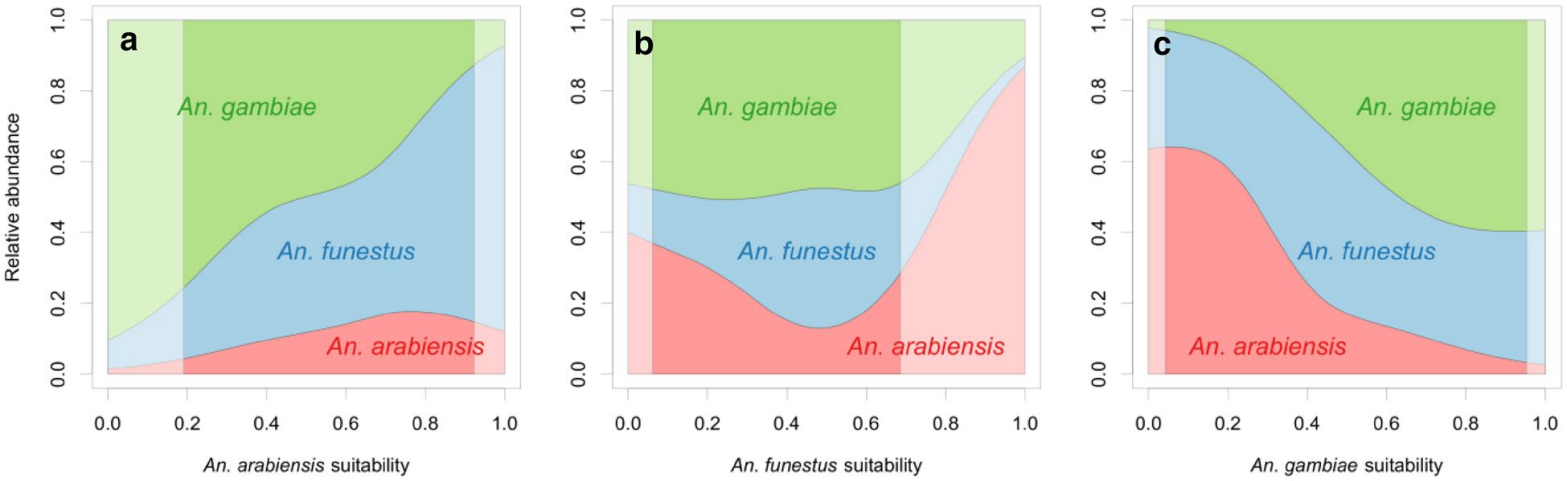
Conditional effect plots for a multinomial generalized additive model of mosquito relative abundance. Plot **a** shows *An. arabiensis,* plot **b** shows *An. funestus* and plot **c** shows *An. gambiae*/*An. coluzzii*. Each plot illustrates the relationship between the predicted relative abundance of the three species and predicted habitat suitability (from Sinka et al. [15] on the logit-scale, rescaled to the unit interval) for each vector. In each plot the habitat suitability values for the other two species are held at their mean values in the dataset: *An. arabiensis* 0.568; *An. funestus* 0.450; *An. gambiae/An. coluzzii* 0.606. The predictions are faded out where the suitability scores are outside the 95 % quantiles of the dataset, where the model had less data with which to estimate the curves and the predictions are therefore less certain (i.e. the suitability scores of the species in question for all of the ratio records except the 2.5 % of datapoints with the lowest suitability scores, and the 2.5 % with the highest, all fall within the* full-colour* region)

The relationship is more clearly illustrated in the effect curves shown in Fig. 2. Each panel shows the expected ratio of the three vectors changing along a gradient of modelled environmental suitability for each species. The plots should be evaluated with the caveat that there is uncertainty, particularly at the extremes of suitability, which is difficult to represent graphically with multinomial data, but is represented here by fading out those predictions where the suitability scores are outside the 95 % quantiles of the dataset (i.e. where the model had less data with which to estimate the curves and are, therefore, less certain). In addition, note that the actual predicted proportions for a given pixel will be a combination of all three of these curves plus the values of the three suitability layers (a four-dimensional plot with three colours would be required to represent the full modelled outcome). However, the trends of the relationship are clear. For example, as the predicted environmental suitability for *An. arabiensis* increases from left to right across the plot, the relative abundance of *An. arabiensis* also increases (although there is a slight dip at very high values where uncertainty is high) (Fig. 2a). The plot also indicates that habitats potentially highly suitable for *An. arabiensis* may also be highly suitable for *An. funestus* but are considerably less so for *An. gambiae*/*An. coluzzii*. Considering *An. gambiae*/*An. coluzzii* (Fig. 2c), again there is a relatively clear picture, and one that corresponds to that of the *An. arabiensis* plot. As the predicted environmental suitability of *An. gambiae*/*An. coluzzii* increases, the relative proportion/abundance of *An. gambiae*/*An. coluzzii* also increases, at the expense of *An. arabiensis*. *Anopheles funestus* remains less affected by the increasing habitat suitability for *An. gambiae*/*An. coluzzii.* The effect plot for *An. funestus* shows a more complicated picture (Fig. 2b). At high levels of ‘suitability’ (where uncertainty is high) the habitat also becomes highly suitable for *An. arabiensis*, which increases the relative abundance of *An. arabiensis* considerably, at the expense of both other species. This again seems to indicate some level of interaction occurring between *An. arabiensis* and *An. funestus*.

### Post-intervention abundance

As the primary mode of vector control in Africa relies on indoor-based interventions, data were identified for the impact of LLINs and IRS. Again, it must be reiterated: the ‘true’ abundance of all the species may have been reduced after the implementation of these interventions. The models predict the post-intervention *relative* abundance of the species populations and therefore illustrate the *relative* effect of the interventions on the different species.

**Fig. 3:**
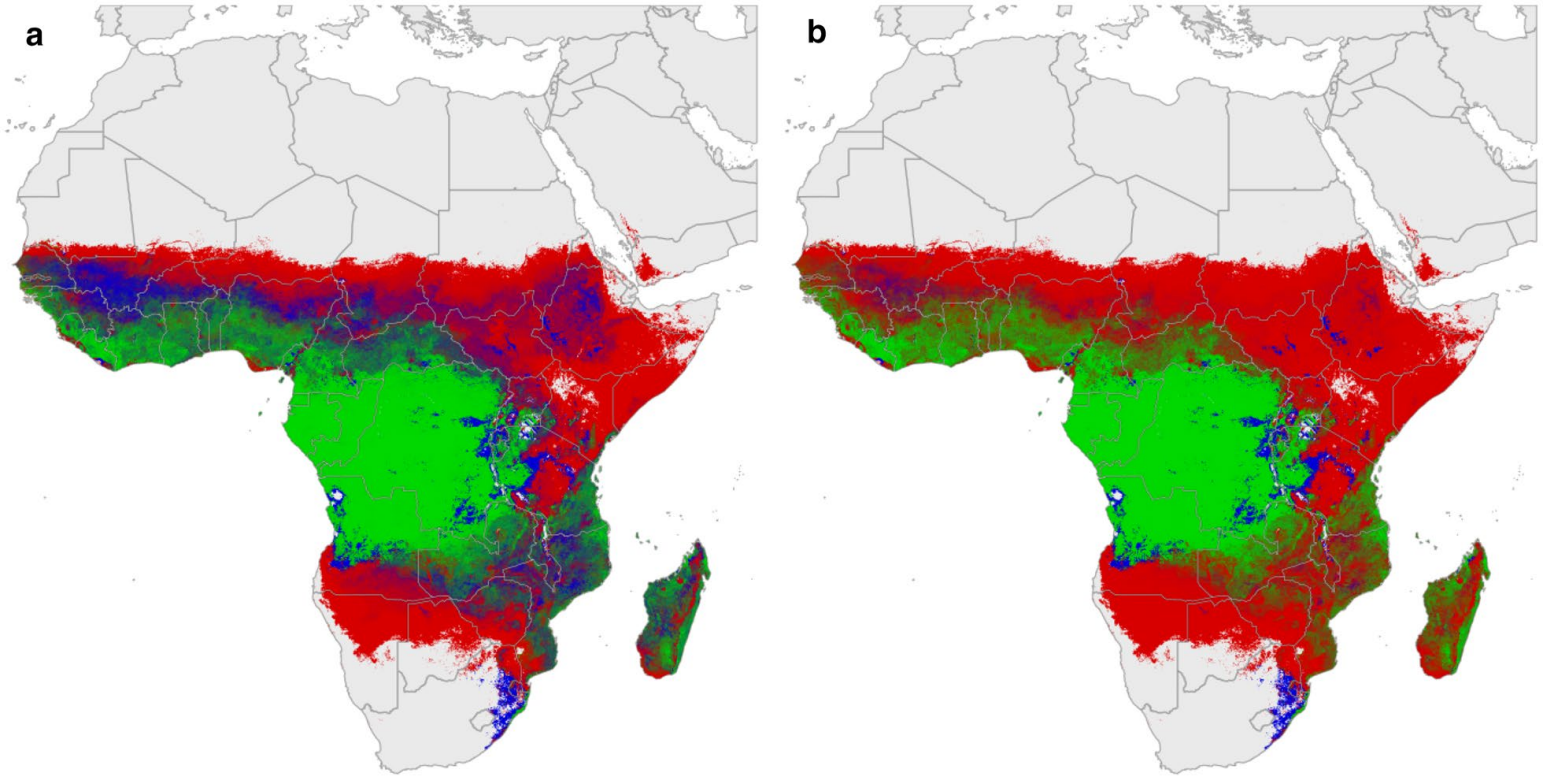
*Red*–*green*–*blue* plots for pre- (**a**), and post- (**b**) intervention (indoor residual spraying) relative abundance. *Red* *An. arabiensis*, *blue* *An. funestus* and *green* *An. gambiae*/*An. coluzzii* with intervening colours indicating the transitioning increasing or decreasing relative abundance between species

**Fig. 4:**
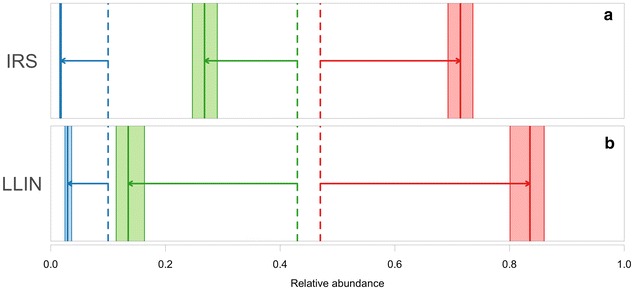
Effect of indoor-based insecticide control on the relative abundance of African vector populations. Plots show the effect of indoor residual spraying (**a**) and long-lasting insecticidal nets (**b**) on the relative abundance of *An. funestus* (*blue*), *An. gambiae*/*An. coluzzii* (*green*) and *An. arabiensis* (*red*), with a hypothetical pre-intervention relative abundance of 0.1 (*An. funestus*), 0.43 (*An. gambiae*/*An. coluzzii*) and 0.47 (*An. arabiensis*), indicated by *vertical dashed lines*. Estimates of post-intervention relative abundances are uncertain and are represented by their 95 % confidence bands (*coloured rectangles*) and maximum likelihood values (*vertical solid lines*). The maximum likelihood estimate of the intervention effect on relative abundance for each species is indicated by the *arrows* linking the pre-and post-intervention estimates

The overall model, comprising species and intervention types and their interactions as well as sampling method, had lower AIC than comparison models. Likelihood ratio tests (using the Chi squared statistic) between these models also indicated that this was the optimal model (df1-2, all p < 0.001). The estimated intervention effect sizes for all species-intervention combinations are shown in Table 1. Although the data are relatively limited so uncertainty levels are high, the model outputs were relatively robust: the model explained 39.7 % of null deviance in abundance and the correlation between predicted and observed post-intervention abundance was 0.684. A clear effect is seen in all the post intervention plots; for IRS, the relative abundance of *An. funestus* and *An. gambiae*/*An. coluzzii* is reduced in favour of *An. arabiensis* (Figs. 3, 4a). This effect is repeated for all indoor-based interventions, with LLINs impacting more strongly on the relative abundance of *An. gambiae*/*An. coluzzii* resulting in a greater increase in the relative abundance of *An. arabiensis* (Fig. 4b), (see Additional file 1: Figure S2).

**Table 1:** Estimated effect of indoor insecticide-based interventions on the abundance of the primary African vector species

	*An. arabiensis*	*An. funestus*	*An. gambiae*/*An. coluzzii*
Indoor residual spraying (IRS)	0.34 (0.32–0.36)	0.038 (0.03–0.04)	0.139 (0.13–0.15)
Long-lasting insecticide-treated nets (LLINs)	0.304 (0.25–0.37)	0.051 (0.05–0.06)	0.054 (0.05–0.06)

Effects are expressed as the median (95 % confidence interval) predicted ratio of post-intervention to control abundances inside houses, per species-intervention combination. Smaller numbers therefore indicate more significant expected reductions

## Discussion

The relative abundance species maps presented here rely on measured relative abundance input data (the reported sample numbers from multi-species collections collated from published studies), but also, and significantly, on the output of species distribution models mapping the distribution of the vectors using a BRT methodology. The BRT species models used a large database of occurrence data and a combination of climatic and environmental variables to define the species’ niche (see Additional file 1: Table S1) and by searching for other locations where such conditions exist, the predicted range of the species was mapped. A probability of presence (equivalent to a habitat stability metric) was calculated depending on how closely the conditions in a particular pixel match those defined as the species’ niche. Therefore, despite a lack of measured relative abundance data in some areas across Africa—notably central Africa, (where the current model output suggests that there is likely to be a higher relative abundance of *An. gambiae*/*An. coluzzii* than for the other species) it is the ‘probability of presence’ in these areas, calibrated using the ‘raw’ relative abundance data, that are compared across the species. This provides a more reliable estimate of relative abundance than would be possible based on the measured relative abundance data alone.

The maps presented here are the only available continent-wide estimates of relative abundance for the primary African vectors of human malaria. These species are globally the most studied and therefore had the most available data for this novel work. They are also highly effective vectors with, for example, a high predominance of endophagy, endophilly and anthropophilly associated with *An. gambiae*, *An. coluzzii* and *An. funestus.* As such they are more likely to have a clear response to control measures that target these behaviours, such as LLINs and IRS. Moreover, the species studied are sympatric across large areas of the sub-Saharan continent. Thus, these were the species deemed both most useful and also most likely to produce accurate outputs in terms of estimating relative abundance.

A literature search suggests that the only other maps currently available examining African malaria vector relative abundance are those of Lindsay et al. [20] who examined *An*. *arabiensis* and *An. gambiae*/*An. coluzzii.* They predicted a much greater distribution of *An. gambiae*/*An. coluzzii* with a narrower band of *An. arabiensis*. This does highlight the point that these are maps of *relative* abundance between the species shown and cannot be used to indicate *actual* abundance. If the presence of *An. funestus* were removed from the current model, for example, the resulting maps may suggest dominance by *An. gambiae*/*An. coluzzii* over *An. arabiensis* similar to that shown by Lindsay et al.

The pre-intervention, multi-species, relative abundance map shown here (Fig. 3a) highlights a much more complex picture of sympatric species interactions than can be seen on previous multi-species maps that rely on overlaying single species distributions (e.g. [21]). *Anopheles arabiensis* and *An. gambiae*/*An. coluzzii* are considered to share many larval site characteristics [22–24] despite some ambiguity in the larval site preference of *An. gambiae*. The identification of the two molecular forms of the species that are now formally named as *An. coluzzii* (previously molecular Form M) and *An. gambiae* (previously molecular Form S) [1] may explain some of this variability in parts of their sympatric range. Indeed, *An. coluzzii* is reported to use larval sites more similar to *An. funestus* (larger, more permanent sites such as swamps and those associated with human activity/irrigation) whereas *An. gambiae* (as it is now, previously molecular Form S) does still appear to share larval site characteristics with *An. arabiensis* (small, temporary sites such as rain puddles), although the evidence for these differences is still far from clear cut [23–25].

Laboratory studies suggest that overall, *An. arabiensis* is detrimentally affected when sharing larval sites with *An. gambiae,* in terms of survival [26, 27] and time to pupation [28], although this is mitigated to some degree at higher temperatures [27]. Indeed, temperature and rainfall are highly influential in defining these species’ distributions [15] and it is these factors that may be striating the species in terms of dominance. *Anopheles arabiensis* larvae are able to survive higher overall temperatures than *An. gambiae*/*An. coluzzii* [27] (but surprisingly not a great deal higher than *An. funestus* [29]) and greater temperature fluctuations than both *An. gambiae*/*An. coluzzii* and *An. funestus* [30]. *Anopheles arabiensis* adults are also more tolerant of drier conditions and able to persist in more arid environments compared to *An. gambiae*/*An. coluzzii* [20] and, therefore, *An. arabiensis* dominates in the drier northern reaches of the shared range of these species. *Anopheles funestus*, by using larger, more permanent larval sites may avoid the issues of high temperature fluctuations or aridity drying their larval habitat and being able to tolerate higher overall temperatures may allow it to dominate in the intermediate climate between the arid conditions exploited by *An. arabiensis* and humid conditions dominated by *An. gambiae*/*An. coluzzii.* However, where *An. gambiae* and *An. coluzzii* are considered separately (and once sufficient data is available, modelling the interaction between these species would prove a valuable application for the models developed here) there is evidence that *An. coluzzii* is more drought tolerant than both *An. gambiae* and *An. arabiensis* [31–33].

Since the maps consider the expected relative abundances in the absence of vector control, estimates of the effect of the main insecticide-based control interventions (IRS and LLINs) on the relative abundances of these species were also produced. These interventions, representing indoor-based application of insecticides, are the control measures most widely used across Africa and have been the cause of significant reductions in malaria transmission across the continent [34–37]. However, by targeting vector species that are either biting or resting indoors, these interventions could differentially affect the relative abundance of various vector species within an area [38, 39]. As such, relative abundance maps may be improved and reflect a more realistic on-the-ground situation by combining them with mapped estimates of intervention coverage, as demonstrated here.

A study examining the effect of IRS and LLINs (as well as anti-malarial drug therapy) on malaria transmission highlighted the greater effect of LLINs in Africa compared to IRS [37]. Moreover, the authors also mapped the resulting changes in malaria transmission across Africa where a notable reduction can be seen in the humid and forested regions of central Africa. *Anopheles gambiae*/*An. coluzzii* are known to dominate in those areas and *An. arabiensis* is rarely, if ever, found [15]. The authors did state that their results do not necessarily relate to the effectiveness for the intervention method but may be a factor of the length of time the intervention has been deployed and the level of coverage. The models presented here however, do suggest that these interventions impact differentially, depending on species. LLINs, more so than IRS, reduce the predicted relative abundance of *An. gambiae*/*An. coluzzii* in favour of *An. arabiensis.*

Examining the relative abundance, however, cannot infer the overall success or failure of a vector intervention programme. The work of Bhatt et al. [37] has highlighted the success of LLINs in reducing malaria transmission in areas within Africa, and consequently, it can be inferred that all relevant vector species have been reduced in their abundance in those areas. This is not apparent simply by examining the relative abundance. Nonetheless, the work presented here may be a significant stepping stone in future calculations of ‘true’ abundance.

The analysis of 575 measurements from 21 prior studies shows an increase in the relative abundance of *An. arabiensis* after the application of these interventions, as expected, and highlights the need to include outdoors measures such as larval source management, baited sugar traps or outdoor space or livestock spraying in the suite of interventions used [7, 40]. A high impact of indoor-based insecticide control on *An. funestus* was also seen, with a lesser effect on *An. gambiae*/*An. coluzzii* and little effect on *An. arabiensis*. Despite similarities in adult behaviour between *An. gambiae*/*An. coluzzii* and *An. funestus* (highly anthrophilic, endophilc and endophagic species), *An. funestus* is known to have a greater vulnerability to indoor applications of insecticides. Gillies and De Meillion [4] state ‘…*funestus* shows a closer adaptation to human dwellings than any other African anopheline. In many areas it spends the greater part of its adult life in houses, which has made it one of the most vulnerable of species to attack with residual insecticides’. This statement is clearly borne out here. Such a clear response to indoor insecticides makes the emergence of insecticide resistance in this species all the more likely (e.g., [41]). As insecticide resistance becomes more widespread, this factor will need to be incorporated into mapping exercises such as those presented here.

## Conclusion

Information on the relative abundance of vector species is vital to inform models that predict the impact of suites of interventions on transmission in different settings. The estimates provided here will allow models to account for variation in the relative abundance of the primary vectors in Africa. Moreover, the methods developed here can be applied to a broader range of vectors, or indeed, any other sympatric species, including the component *An. gambiae* species: *An. coluzzii* and *An. gambiae*, once sufficient data for these are available.

### **Availability of supporting data**

The occurrence data set supporting the results reported in this manuscript are available via the Malaria Atlas Project (MAP) website [16]. Additional intervention data are available on request.

## Additional file

10.1186/s12936-016-1187-8 Data location plot, pre and post-intervention (LLINs) relative abundance red–green–blue plots and climatic variables identified by the BRT model. The document provides a map detailing the location of the data used in the work presented as well as additional red–green–blue plots for the pre and post-intervention (by LLINs) relative abundance of the three vectors. Also included is a table listing the top five climatic variables predicted by the BRT model as most influential in describing habitat suitability for each of the three vectors.

## Abbreviations

LLIN: long-lasting insecticidal nets
IRS: indoor residual spraying
EIR: entomological inoculation rate
HBR: human biting rate
SDMs: species distribution models
DVS: dominant vector species
BRT: boosted regression tree
GAM: generalized additive model
AIC: akaike information criterion value
MAP: malaria atlas project
