# Supplementary material for: Modelling the relative abundance of the primary African vectors of malaria before and after the implementation of indoor, insecticide-based vector control
Source: Malar J. 2016 Mar 5;15:142. doi: 10.1186/s12936-016-1187-8 (PMC4779559; doi:10.1186/s12936-016-1187-8)
Supplement: Supplementary file 1 — 10.1186/s12936-016-1187-8 Data location plot, pre and post-intervention (LLINs) relative abundance red–green–blue plots and climatic variables identified by the BRT model. The document provides a map detailing the location of the data used in the work presented as well as additional red–green–blue plots for the pre and post-intervention (by LLINs) relative abundance of the three vectors. Also included is a table listing the top five climatic variables predicted by the BRT model as most influential in describing habitat suitability for each of the three vectors. [file 12936_2016_1187_MOESM1_ESM.docx]

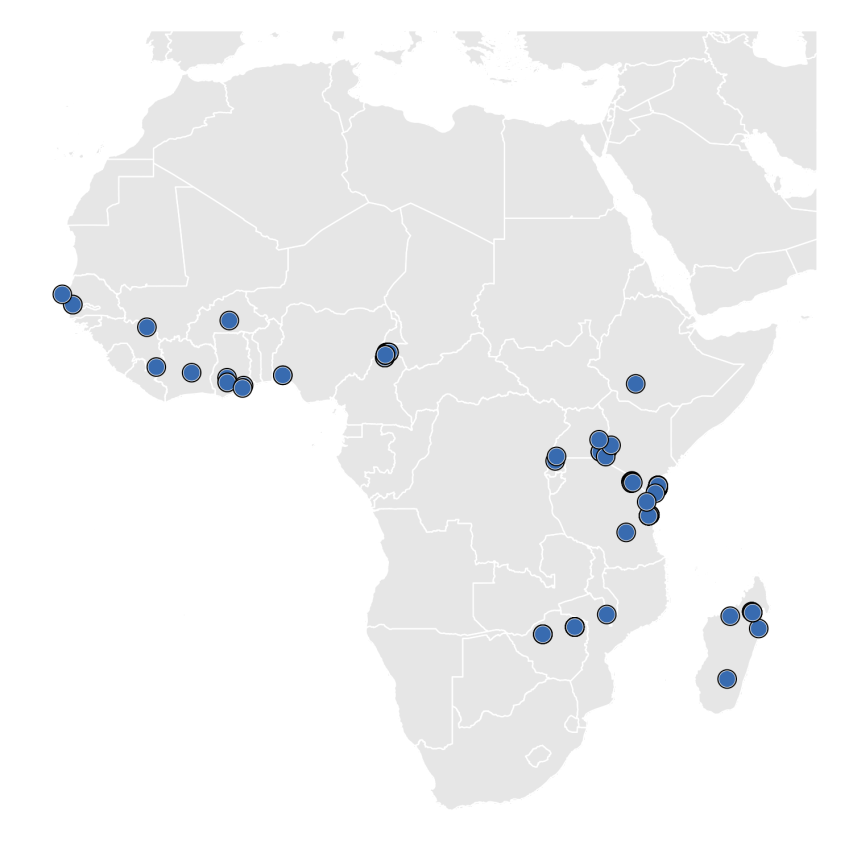


Figure A1: Data location used in the generalised additive model (GAM)


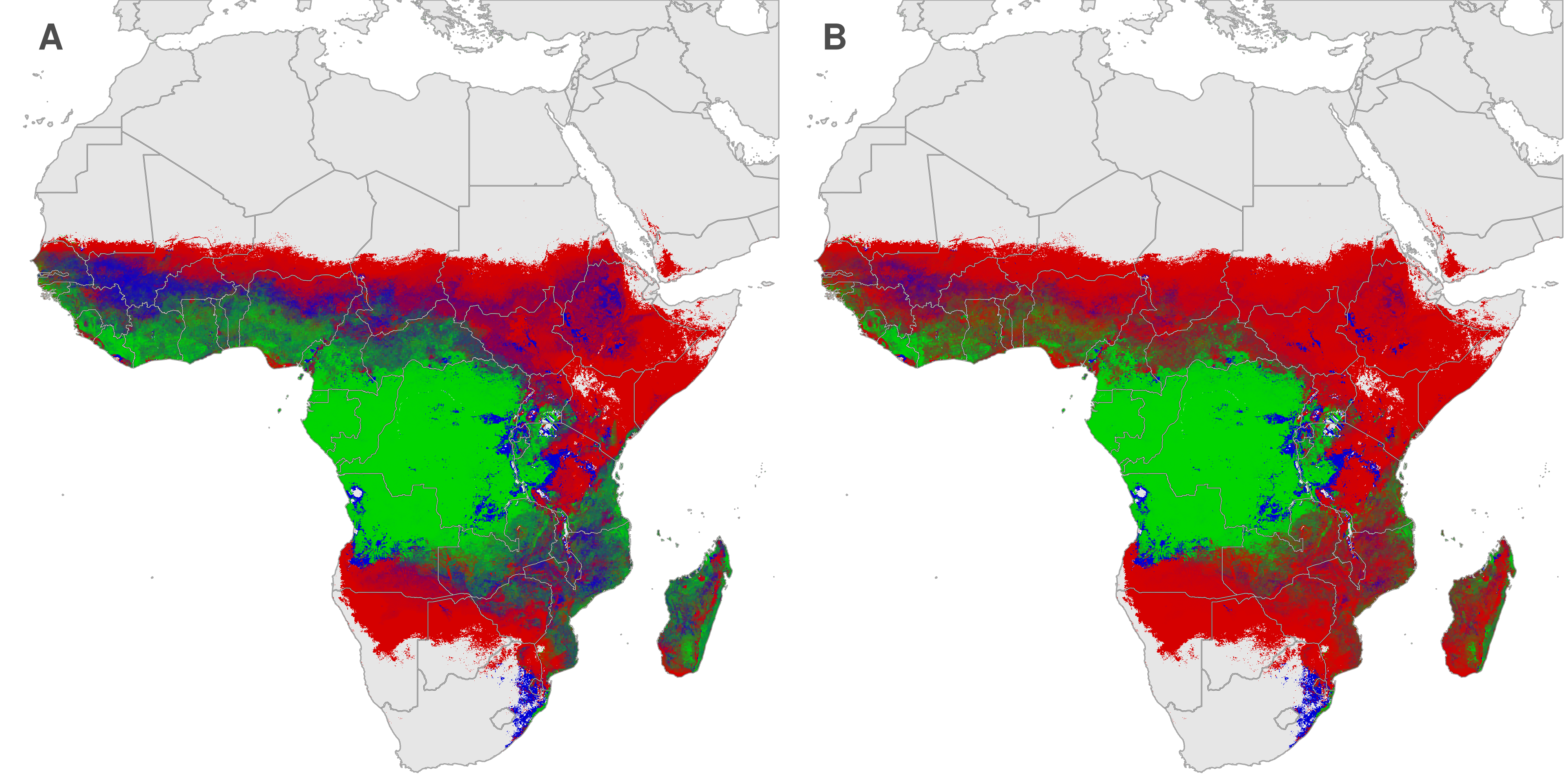


**Figure A2:** **Red-green-blue plots for pre- (A), and post- (B) intervention (Long lasting insecticidal nets) relative abundance.** Red = *An. arabiensis*, blue = *An. funestus* and green = *An. gambiae* with intervening colours indicating the transitioning increasing or decreasing relative abundance between species.

**Table A1:** **The top five environmental/climatic variables selected by the BRT for predicting the habitat suitability of *An. arabiensis*, *An funestus* and *An. gambiae*/*An. coluzzii*.** Numbers in parenthesis after the species name indicate the number of occurrence points used in the model.

| Species | Environmental variables |
| --- | --- |
| *An. arabiensis* (1196) | \| 1 \| NDVI (phase of the annual cycle) \| \| --- \| --- \| |
|  | \| 2 \| Prec (amplitude of the bi-annual cycle) \| \| --- \| --- \| |
|  | \| 3 \| LST (phase of the annual cycle) \| \| --- \| --- \| |
|  | \| 4 \| Prec (max.) \| \| --- \| --- \| |
|  | \| 5 \| MIR (phase of the annual cycle) \| \| --- \| --- \| |
| *An. funestus* (919) | \| 1 \| Prec (max.) \| \| --- \| --- \| |
|  | \| 2 \| NDVI (mean) \| \| --- \| --- \| |
|  | \| 3 \| Prec (amplitude of the bi-annual cycle) \| \| --- \| --- \| |
|  | \| 4 \| MIR (mean) \| \| --- \| --- \| |
|  | \| 5 \| NDVI (amplitude of the annual cycle) \| \| --- \| --- \| |
| *An. gambiae*/*An. coluzzii* (1443) | \| 1 \| Prec (mean) \| \| --- \| --- \| |
|  | \| 2 \| Prec (max.) \| \| --- \| --- \| |
|  | \| 3 \| DEM \| \| --- \| --- \| |
|  | \| 4 \| Prec (amplitude of the bi-annual cycle) \| \| --- \| --- \| |
|  | \| 5 \| LST (min.) \| \| --- \| --- \| |

NB: NDVI: Normalized Difference Vegetation Index; Prec: Precipitation; LST: Land Surface Temperature; MIR: Middle Infrared; DEM: Digital Elevation Model
